# Inaugural Editorial: Curating the Catalog of Knowledge About Gambling and Gambling Problems

**DOI:** 10.1007/s10899-025-10376-0

**Published:** 2025-02-05

**Authors:** James P. Whelan

**Affiliations:** Tennessee Institute for Gambling Education & Research, The University of Memphis, Memphis, US

Editorial History

As I assume the honor of being the editor of the *Journal of Gambling Studies*, I wish to both commend the contributions of my predecessors and briefly describe where we came from before describing our future. In 1985, Henry Lesieur, the founding Editor-in-Chief, wrote, “This journal is about gambling… (p.3)” to open the journal’s first issue. This phrase seemed born from a sense of purpose, as well as an experience of frustration. Lesieur provided a convincing rational for justifying a gambling-specific academic journal – there was a long history of peoples’ involvement in gambling and a complicated history where the harms of legal and social gambling needed to be addressed with the same academic rigor as other social harms. He highlighted that legal access to gambling was expanding in the United States (US) and impacting millions of people, and the recognition that the harms of gambling led to the American Psychiatric Association’s recognizing Pathological Gambling as a psychiatric diagnosis in 1980. Lesieur gave voice to the frustration that the mental health community and addiction journals ignored gambling problems and research. Basically, the journal began with a sense of urgency regarding the need to increase accessibility and legitimacy of evidence-based research of the Gambling Disorder.

Lesieur succinctly declared that the new journal would be focused on gambling and gambling pathology. He then invited submissions on treatment for those who experienced harm. A review of the first 5-years reveals that the journal stayed true to this call. In 1990, William Eadington joined as a co-editor and together with Lesieur introduced several meaningful shifts to this periodical (Eadington & Lesieur, 1990). For one, the title the *Journal of Gambling Behavior* was changed to *Journal of Gambling Studies*. Such changes were said to “continue to develop a broader perspective and to allow for greater emphasis on public policy research, economic and political analysis of gaming legislation, and of commercial gaming industry, gambling and the law, industry studies and social science analysis in general (p.110).” A clear subtext to this editorial, although never stated, was the need for the journal to remain gambling neutral, with papers about the benefits of gambling welcomed. A broadening scope was evident for much of the next decade, although papers on gambling problems and pathology continued to dominate.

In 1997, Howard J. Shaffer became editor-in-chief (Shaffer, 1997). His editorial announcement reiterated the journal’s rationale and reaffirmed a commitment to the aims and scope defined by Eadington and Lesieur. Shaffer reiterated the observation that legal gambling accessibility in the US continued to expand. He called for the journal to “guide America through the complexities associated with gambling (p.4)”, and he restated the value of contributions across academic fields. There was no doubt that, like Lesieur, Shaffer understood that gambling problems were central to the journal’s purpose as he called for “understanding the biology, sociology, and psychology of intemperate gambling (p.4)”, to “better understand the nature of addiction… (p. 4).” Shaffer’s efforts to shift the journal were revealed over the next few years as he discussed a strong commitment to editorial efficiency, the peer review process, and the growth of the journal through dedication to science (Shaffer et al., 1998). He voiced that the journal had grown to 88 unique submissions in 1988, which prompted the addition of associate editors. He also encouraged direct debates on controversial topics through a mechanism he called the Exchange section (Shaffer & Bethune, 1999). With this growth came an increase in the publication of empirical studies completed outside North American. After several years without special issues, Shaffer’s editorship included special issues on critical topics, such as neurobiology, socioeconomic impact, and comorbidity.

Jon Grant’s tenure as editor-in-chief began in 2003 and ended in 2024. Grant was quiet about his editorial position. In many ways, during Grant’s tenure, the journal returned to Lesieur’s primary focus on understanding gambling behavior and addressing or treating gambling pathology. Fewer papers from disciplines outside of psychology, mental health, and psychiatry appeared to be published. Exchanges on controversial topics and special issues were discontinued or minimalized. At the same time, reading the papers published across Grant’s tenure indicates some evolution of the journal. For example, there was an increase in the proportion of empirical papers and reviews from outside North America, as well as a tremendous growth in submissions. In 2007 the journal received over 100 submissions for the first time. The numbers appear to climb slowly but steading. By 2024 submissions were almost three times the number achieved in 2007. The field is greatly indebted to Jon Grant for single-handedly managing a flourishing journal and managing a review process for this ever-growing number of papers. His typically silent dedication to effectively shepherding this journal for over two decades is amazing. His influence on the entire field cannot be denied; we all owe him our deepest gratitude.

Editorial Future

As Journal of Gambling Studies begins its 41st year in publication, I am honored to be selected as the next Editor-in-Chief. My first goal was to summarize the journal’s editorial history and give credit to previous editors. Henry Lesieur, William Eadington, Howard Shaffer, and (now) Jon Grant have all been impactful scholars who should be recognized for their dedicated, inspiring, and brilliant contributions to this journal. We sometimes mistake journal editing for an administrative or gatekeeping task. Having submerged myself in the 40 years of this journal’s indexes, I cannot help but appreciate the need for an editor to facilitate the scientific rigor of its subject. As a scientist-practitioner who has researched and provided treatment for gambling disorder for more decades that overlap these years of indexes than I sometimes care to admit, my scholarship and the successes of the clients at my treatment clinics have been directly benefited from this journal’s editorial vision.

The second reason for my review was to anchor my vision of how to carry forward the journal’s purpose. From Lesieur through Grant, the *Journal of Gambling Studies* has been dedicated to advancing the knowledge of gambling behavior from risk-taking to gambling disorder. The journal has reliability reaffirmed a commitment to a high standard of empirically based efforts and has welcomed a diversity of perspectives. The aims include publishing manuscripts that can inform policy, practice, understanding while also contributing to a global understanding of the complexities associated with the harms caused by gambling. My hope as editor is to build a comprehensive and valuable catalog of new knowledge that serves our different stakeholders including **researchers**, **service providers**, **regulators**, and the **gambling industry.**

Looking over the past 40 volumes of this journal provided a context for understanding how to continue its success while at the same time addressing the needs of our different stakeholders. The first lesson is that this journal has continued to garner an always-increasing number of submissions. To be prepared to address this growth, three outstanding researchers will join the journal as Associate Editors: Meredith K. Ginley, (East Tennessee State University), Andrew (Hyounsoo) Kim (Toronto Metropolitan University), and Rory A. Pfund (University of Memphis). My choice to select early and mid-career academics was intentional. This trio are productive contributors to the field, committed to this journal, and representative of the talent and knowledge we need to carry forward the future science needed to better understand gambling. They will be closely involved in the process of curating this catalog of new knowledge and rebuilding the editorial board to blend the field’s history with an emphasis on the expanding range of gambling-related research questions. Together we will work to continually improve this process. To gain review efficiency, we will institute a pre-review process, complete with checks and balances, that should help us adhere to the journal’s scope and aims, and respect reviews time and efforts.

Finally, the success of this journal requires commitment from the field. One form of commitment is the submission of quality papers that mobilize our knowledge. Since 1985 the number of other outlets that welcome gambling research has grown. We would like you to continue contributing to this journal and make it one of the premier outlets for new gambling-related knowledge. The other important form of commitment is to volunteer as a reviewer. Diving into the journal’s submission portal revealed to me the challenges in securing reviews. The journal’s success comes from us providing each other with thoughtful reviews aimed at helping authors shape their message and sharpen how they present their data. The hope is that you will join the Associate Editors and I as we commit to the success and value of this journal. In advance, thank you for your trust and support as we revise our review request process to ensure we use reviewers’ time most effectively and efficiently. As experts with specialized knowledge invaluable to our science, please know your assistance is needed and appreciated.

James P Whelan

Editor in Chief

## References

[CR1] American Psychiatric Association. (1980). *Diagnostic and statistical Manual of Mental disorders* (3rd ed.). Author.

[CR2] Eadington, W. R., & Lesieur, H. R. (1990). A New Title and co-sponsorship of the Journal. *Journal of Gambling Studies*, *6*(2), 109–111. 24242852 10.1007/BF01013491

[CR3] Lesieur, H. R. (Ed.). ’s Introduction. Journal of Gambling Behavior, 1(1),3–7.

[CR4] Shaffer, H. J. (1997). Editorial: Images, versions, and vision: The Journal of Gambling studies enters another season. *Journal of Gambling Studies*, *13*(1), 3–5.

[CR5] Shaffer, H. J., & Bethune, W. (1999). Journal of Gambling studies Editorial characteristics 1997–1998. *Journal of Gambling Studies*, *15*(3), 179–180.

[CR6] Shaffer, H. J., McNamara, E., & Bethune, W. (1998). The JGS welcomes new associate editors. *Journal of Gambling Studies*, *14*(4), 307–308.

